# A review of the clinical utility of serum S100B protein levels in the assessment of traumatic brain injury

**DOI:** 10.1007/s00701-016-3046-3

**Published:** 2016-12-12

**Authors:** Eric Peter Thelin, David W. Nelson, Bo-Michael Bellander

**Affiliations:** 1 Division of Neurosurgery, Department of Clinical Neurosciences, University of Cambridge, Cambridge Biomedical Campus, Cambridge, UK; 2 Department of Clinical Neuroscience, Karolinska Institutet, Stockholm, Sweden; 3 Neurosurgical Research Laboratory, Karolinska University Hospital, Building R2:02, S-171 76 Stockholm, Sweden; 4 Division of Perioperative Medicine and Intensive Care (PMI), Section Neuro, Karolinska University Hospital, Stockholm, Sweden; 5 Department of Physiology and Pharmacology, Section of Anesthesiology and Intensive Care, Karolinska Institutet, Stockholm, Sweden; 6 Department of Neurosurgery, Karolinska University Hospital, Stockholm, Sweden

**Keywords:** S100B, Traumatic brain injury, Outcome, Monitoring, Screening, Biomarker, Serum, Humans

## Abstract

**Background:**

In order to improve injury assessment of brain injuries, protein markers of pathophysiological processes and tissue fate have been introduced in the clinic. The most studied protein “biomarker” of cerebral damage in traumatic brain injury (TBI) is the protein S100B. The aim of this narrative review is to thoroughly analyze the properties and capabilities of this biomarker with focus on clinical utility in the assessment of patients suffering from TBI.

**Results:**

S100B has successfully been implemented in the clinic regionally (1) to screen mild TBI patients evaluating the need to perform a head computerized tomography, (2) to predict outcome in moderate-to-severe TBI patients, (3) to detect secondary injury development in brain-injured patients and (4) to evaluate treatment efficacy. The potential opportunities and pitfalls of S100B in the different areas usually refer to its specificity and sensitivity to detect and assess intracranial injury.

**Conclusion:**

Given some shortcomings that should be realized, S100B can be used as a versatile screening, monitoring and prediction tool in the management of TBI patients.

## Introduction

Traumatic brain injury (TBI) is a common cause of death and disability, primarily in the young but increasingly among the elderly [153]. The injury panorama stretches from the severely injured, unconscious patients in need of neuro-intensive care to the more common mildly injured patients, sometimes without any visual lesions. Many survivors, even from seemingly mild injuries, may suffer from permanent disabilities and be in need of long-term rehabilitation with high costs for society [56].

TBI is a complex disease and may change symptomatology over time [102]; it is heterogenic in nature and may contain a plethora of different hemorrhagic and non-hemorrhagic injures, both inside and outside the brain parenchyma. At admission to the hospital, the physicians often rely solely on a neurological examination and a computerized tomography (CT) scan, as other more advanced radiological options are unavailable in the acute care setting and monitoring tools are available only in specialized neuro-intensive care units (NICUs). Consequently, the assessment methods are often limited, and better surrogate markers of brain injury have been sought to help the treating clinician. In many fields of medicine, biological markers (“biomarkers”) of injury have been introduced. A biomarker is defined as “A characteristic that is objectively measured and evaluated as an indicator of normal biological processes, pathogenic processes, or pharmacologic responses to a therapeutic intervention” [54]. It may be proteins, functioning as surrogate indicators of injury, such as troponin-T in the screening for myocardial infarction [60] and D-dimer for deep vein thrombosis [198]. While a number of potential markers of brain tissue fate (i.e., increasing levels of the specific markers indicating affected or dying cells) exist, the most studied protein biomarker of brain injury is S100B.

The first human TBI study of S100B’s value as a serum biomarker of brain injury assessment was published by Ingebrigtsen and coworkers in 1995 [75], although increased S100B levels in cerebrospinal fluid (CSF) following various neurological disorders had been previously described in patients by Sindic et al. in 1982 [165]. Later on, S100B was shown to be sensitive enough to detect and assess different traumatic intracranial lesions, including cerebral contusions [144], subdural hematomas and traumatic subarachnoid hemorrhages [152], as well as epidural hematomas [189].

While several reviews currently exist, highlighting the role of S100B in both mild [193] and moderate-to-severe TBI [104], new aspects have evolved in this field such as the finding of S100B transportation through the newly discovered glymphatic system [141], implementation of the Scandinavian guidelines for TBI incorporating S100B [194] and improved kinetic modeling of S100B release from the injured brain [42].

This review aims to give the clinician a comprehensive overview of the utilities of S100B in the assessment of brain injury and may thus be used as a guide to interpret serum S100B samples in the treatment of both mild and moderate to severe TBI patients.

## The protein S100B

The protein S100 was originally isolated from bovine brain almost 50 years ago and got the name from its 100% solubility in a saturated ammonium sulfate solution [108]. S100 is a relatively small protein, 9–14 kDa, present physiologically mainly as different homodimers [36]. It belongs to a family of intracellular, calcium-binding proteins predominantly present in mature, perivascular astrocytes, but is also present to some extent in other cells in the central nervous system (CNS), including oligodendrocytes, neural progenitor cells and certain neuronal populations [39, 172]. In the CNS, the protein exists mainly as the homodimer S100BB or heterodimer S100AB [58, 76]. Together, these proteins make up the levels of what is usually referred to clinically as “S100B” (or sometimes “S100B Total”). Several other proteins of the S100 family exist, whose functions have been previously described in several review articles [37, 38, 84].

Ideally, a biomarker of brain injury should fulfill the following criteria according to prominent authors in the field [84, 130] (Table 1). Currently, no biomarker fulfills all the criteria, but as S100B is the most studied, we are learning how to interpret and assess it in different situations, thus minimizing and avoiding potential limitations.

**Table 1:** Suggested properties for biomarkers in traumatic brain injury

Demonstrate a high sensitivity and specificity for brain injury
Exhibit a passive release from the central nervous system (CNS) without any stimulated active release
Lack specific effects on CNS cells interfering with the initial injury
Useful to stratify patients by severity of injury and provide information about injury mechanisms
Have a rapid appearance in accessible biological fluids and an unlimited passage from the brain
Have well-defined bio-kinetic properties and monitor progress of disease and response to treatment
Predict functional outcome

## Functions of S100B

S100B has several properties, previously reviewed [36–38, 49, 106, 196]. Intracellularly, S100B is a normal part of calcium hemostasis, thereby transferring signals from second messengers [64]. S100B is also involved in cell differentiation and cell cycle progression [158], and it has been shown to inhibit apoptosis if applied in experimental conditions [25]. Extracellularly, in both normal physiology and during traumatic conditions, administered S100B promotes neurogenesis [1, 57] and neuronal plasticity [103, 120], performs neuro-modulating actions and enhances processes involved in memory and learning [43, 83].

However, the effect and physiological functions of S100B have been shown to be concentration dependent, where lower concentrations (nanomolar levels) are beneficial and higher concentrations (micromolar levels) are correlated to harmful effects [154, 196]. Escalating extracellular levels of S100B have been shown to result in neuronal dysfunction or cell death because of an inflammatory response that stimulates astrocytes and microglia to recruit and produce proinflammatory cytokines with a subsequent increase of the extracellular levels of calcium and activation of nitric oxide, with harmful effects [69, 89].

The different effects of S100B have been suggested to depend on the Receptor for Advanced Glycation End-products (RAGE), which is upregulated by S100B levels and may cause proinflammatory gene activation [38], although much is still unknown about how S100B exerts its biochemical properties.

## Release and elimination of S100B

In vitro studies reveal that astrocytes, when affected by trauma or metabolic distress [47], will release stored S100B, which has been measured extracellularly as fast as 15 s after a lesion is established [200]. The S100B mRNA concentration, as a sign of ongoing intracellular synthesis of the protein, will also increase shortly after injury [68]; thus, the concentrations measured in blood are from both secreted and newly synthesized origins. However, looking at the volumes of synthesized mRNA, the majority of the measured S100B in bodily fluids seems to come from dead or dying cerebral tissue.

It is known that the concentration of S100B in the CSF could be up to 100× higher than in serum [137]. A majority of the S100B is presumably released directly from the CSF to serum through the arachnoid villi, as the ratio between the CSF:serum of S100B is correlated, especially early after the TBI [51]. When the patient suffers from TBI, the blood-brain barrier (BBB) is disrupted, causing a leakage of proteins from the CSF with subsequent cerebral deterioration and edema formation [100]. The ratio of albumin between CSF:serum (Q_A_) is often used to quantify the degree of BBB disruption [184]. Some authors claim that S100B is released into the serum through the disrupted BBB [80, 81, 99]; thus, it is a good marker of BBB permeability [20, 97]. However, the studies supporting S100B as a marker solely for BBB integrity are limited in sample size, use osmotic or chemo-therapy in non-TBI patients to disrupt the BBB and are not focused on the complex BBB disruption present following traumatic conditions. Studies that focus on TBI patients do not show any correlation between a disrupted BBB, using Q_A_, and the peak serum levels of S100B [14] or by using a ratio of the CSF and serum S100B compared to Q_A_ [87], hence indicating a better correlation between actual injury and S100B levels and not to the degree of BBB disruption.

Furthermore, a route between the para-arterial influx, interstitial fluid of the brain, cerebrospinal fluid and venous outflow has recently been discovered, entitled the glymphatic system because of the connection between glial cells and aquaporin-4-dependent paravascular pathways (mimicking a lymphatic drainage from the brain) [71]. It has recently been shown that this system probably plays a very important role in the outflow of S100B from the brain, which could explain the discrepancies found in TBI studies looking at correlations between BBB integrity and S100B release [141]. Recent findings in animal models suggest that S100B, as well as the biomarkers neuron-specific enolase (NSE) and glial fibrillary acidic protein (GFAP), are released through the glymphatic drainage, independent of BBB permeability [141]. If this is confirmed, it constitutes several challenges to the interpretation of the serum levels of protein biomarkers in humans. First, the recently discovered glymphatic system is, so far, impossible to monitor in vivo in humans, and no surrogate marker to assess the glymphatic system exists today. This will make it difficult to evaluate whether the glymphatic system plays the same role in humans as in animal brain biomarker release. Second, in studies from the Nedergaard group that discovered the glymphatic system [70, 71], the aquaporin-4-containing podocytes of perivascular astrocytes depolarize for up to 4 weeks following experimental traumatic brain injury, resulting in a disrupted glymphatic clearance. In theory, this might also affect the biomarker release from the injured brain in humans. Third, the glymphatic drainage has been shown to be affected if CSF is drained by pharmaceutical compounds as well as if sleep is affected. Draining the CSF and sedating patients (possibly similar to sleep) are two common treatment modalities in the NICU for severely brain injured patients to maintain adequate intracranial pressure. Thus, different actions in the NICU will have an effect on the release of S100B in serum.

Systemically, S100B has been shown to be excreted and eliminated 100% through the kidneys [195]. Patients with renal failure have higher baseline S100B levels compared to healthy controls [107], which should be taken into consideration when assessing S100B in these patients. However, the mild to moderate kidney impairment often seen in trauma patients has not been shown to significantly affect S100B levels in serum [79].

In summary, S100B is released from the CSF to serum, but this drainage could be affected by the glymphatic clearance as well as the BBB permeability. Therefore, although it is seemingly passive, it may in theory be altered by different factors.

## Extracranial sources of S100B

There are several known extracerebral sources of protein S100B, such as Langerhan’s cells, adipocytes, epithelial cells, cardiac and skeletal muscle cells, and chondrocytes [58]. According to researchers involved in the Human Protein Atlas, S100B mRNA is only 1.44 times more expressed in the cerebral cortex compared to adipose tissue, the second most common tissue containing S100B [166]. This could be compared with GFAP mRNA, which is 374.81 times more common in the brain compared to adipose tissue, the second most common source for that protein as well. Notably, GFAP-brain specificity is only “beaten” by oligodendrocytic myelin paranodal and inner loop protein (OPALIN), with mRNA 541.99 times more common in the brain than in any other analyzed tissue [166]. Patients who suffer from trauma, presenting multiple injuries to the thorax, extremities and abdominal organs without any identified damages to the central nervous system, have been shown to exhibit elevated S100B concentrations in serum [7, 138, 155, 190]. However, S100B released from an extracerebral origin appears to have a faster clearance than S100B released from the CNS [33, 134, 157], while some authors suggest a very limited contribution from extracranial sources [139]. In our group, we have also seen that extracranial trauma contributes independently to increased serum levels of S100B [182]. When conducting a sliding window over time, we found that the early peak and values <12 h after trauma are poorly correlated to outcome [180]. After 12 h there is an increase in relations to outcome, which peaks around the end of day 1 [179], which is congruent with the kinetic modeling where the main injury peak is found at 27 h [42]. The early or near immediate peak is assumed to be of extra-cranial origin as it is correlated to multi-trauma and, in contrast to the gamma-variate kinetics of the main peak, appears to follow a first-order kinetic with rapid decay [42].

In summary, while possible extracranial sources should be taken into consideration when assessing TBI patients [135], repeated sampling after 12 h should exclude several of these confounding factors. This, however, has implications for the use of S100B as an early warning sign in mild TBI.

## Half-life of S100B

It is difficult to assess the half-life of S100B in serum after TBI since there will be a continuous release of the protein from the damaged brain and perhaps a contribution from the CSF due to a disruption of the BBB [81], outflow through the glymphatic system [141] and ongoing cerebral synthesis and/or active secretion [47, 68]. The half-life of S100B has been shown to be in the range of 60 to 120 min in patients with TBI [73, 77] and 90 min in patients suffering from malignant melanoma [48]. Another more controlled method makes it as short as 25 min [79], calculated from patients undergoing coronary artery bypass grafting not suffering from any evident brain injury.

In aggregate, the net amount of S100B in serum will thus be the summed effect of the rate of influx and the rate of elimination. As the serum half-life of S100B is short, an extended elevation of S100B indicates an ongoing influx. We have recently shown this to be well fitted to the gamma variate function [42] in patients with TBI. The peak and area under the curve of the increase appear to correspond to the extent of the damage sustained [179].

## Assays to analyze S100B

There are several available assay kits to measure S100B that are used in clinics and laboratories. An advantage of S100B is that it is stable and relatively unaffected by storing, changes in temperature and freeze-thaw cycles [146], which greatly facilitate the handling of the protein and reliability of the analyses. Another advantage with S100B is that it is not affected by hemolysis in the sample [13], making it a robust sample to use in the acute setting. ELISA has become the gold standard for measuring S100B in laboratories, and kits are commercially available from several different manufacturers (Sangtec, Italy; CanAG Diagnostics, Sweden; Syn X Pharma Inc. Nanogen, USA, to name a few). Unfortunately, ELISA assays take 4–6 h to run and generally present higher inter- and intra-coefficients of variation (CVs) compared to clinical assays, resulting in a worse functional sensitivity of the devices [167], making them difficult to use in the clinic.

In clinical settings, the two most frequently used systems are the quantitative automated luminometric immunoassay LIAISON-mat S100 system (Diasorin, Sangtec, Italy) and the electrochemiluminescence immunoassay (Elecsys S100B®; Roche Diagnostics, Penzberg, Germany). The LIAISON system was primarily designed to detect and screen for S100B in malignant melanoma and other tumors and is thus not designed for quick analyses [116]. The Elecsys system and the new automated Cobas® system from Roche Diagnostics are increasing in popularity since they take only 18 min to run a serum sample, making it more suitable for TBI and possible for both emergency room (ER) and NICU use [167].

New point-of-care bedside devices are being developed [122] to be able to swiftly detect elevations of S100B. These kits could be deployed on the battlefield or in ambulances to quickly assess S100B levels above certain thresholds using bio-sensors.

## Clinical use of S100B

A number of review articles have thoroughly analyzed different aspects of the clinical utilities of S100B [10, 19, 44, 84, 88, 91, 104, 159].

S100B from extracranial sources has long been used to monitor the advancement and evaluate the efficacy of therapy of melanocytic tumors [40, 82, 168]. As an interesting note, persons with darker skin have higher serum levels of S100B (median 0.14 μg/l) as compared to persons with brighter skin (median 0.07 μg/l) [15], presumably due to a higher metabolic activity in melanocytes in the former group [6]. This might be of importance when calculating outcome from TBI in patients with darker skin, as higher serum levels may be falsely interpreted as elevated, resulting in an unnecessary CT scan in mild TBI.

A clinical role has also been shown for different cerebral conditions, including stroke [23, 45], global ischemia [115, 195], neurodegenerative disorders such as MS and Alzheimer’s disease [154], spontaneous subarachnoid hemorrhage [109, 123, 156, 175] and cerebral vasospasm [123]. Nevertheless, it is in the field of TBI where it has been mostly studied.

All traumatic cerebral injuries have been shown to increase S100B in serum, but focal injuries, such as cerebral contusions and subdural hematomas, present higher levels as compared to diffuse injuries [67], and contusion volumes have been directly correlated to serum levels of S100B [140, 144]. This further stresses that the amount of tissue affected is much more important than the exact spatial location when assessing brain injury using S100B. Thus, the importance of combining S100B with proper imaging is necessary to visualize anatomical localization of the injury.

However, S100B has been shown to be able to stratify patients by injury severity, as can be seen in studies showing low S100B levels in mild TBI (GCS14-15) and escalating levels in moderate (GCS9-13) and severe (GCS3-8) TBI [67, 152]. The most common CT classification of injury is the Marshall CT classification [101], where S100B has been shown to increase in the focal, more severe, lesions compared to more diffuse injuries [90, 140]. Other classifications of injury include the Rotterdam CT score [98] and Stockholm CT score [117], where an increasing sum is associated with a more unfavorable outcome, also showing a correlation to increased serum levels of S100B as the injury becomes more severe [179]. More novel techniques for image outcome following traumatic brain injury exist; one of them is functional MRI, where levels of S100B in serum have been shown to correlate to the extent of disrupted connectivity in the default mode networks [183].

## S100B to screen for intracranial pathology in adult, mild TBI patients

The vast majority (estimated up to 95%) of all TBI cases present as mild, defined as a Glasgow Coma Scale (GCS) score of 14–15 [29], and are common patients in the emergency room (ER). The physician in the ER is often presented with the challenge of how to assess these patients as they are often intoxicated; up to 50% of mild TBI patients suffer from alcohol intoxication [125] and could have problems remembering what exactly happened as well as whether any post-traumatic symptoms occurred, something that could be difficult even for the sober.

Moreover, some of these patients may develop intracranial hematomas in need of surgical evacuation or intracranial monitoring. To detect these lesions, a computerized tomography (CT) scan of the brain is the preferred radiological choice [170]. However, these should be minimized as they are accompanied by a significant dose of radiation and therefore a slightly increased risk of cancer, and they may even have an effect on cognitive functions later in life [24, 59]. To determine which patients to scan, specific guidelines have been presented, such as the Canadian CT rule [173], the NEXUS-II [111] and the Scandinavian CT guidelines from 2000 [74]. Using the Scandinavian guidelines from 2000, the sensitivity to detect an intracranial lesion is 96%, but the specificity only 53%, similar to the other guidelines [171]. Therefore, even if few lesions are missed using CT scanning, many unnecessary CT scans are performed with excess radiation and costs as a consequence. Furthermore, scanning intoxicated patients may require sedation and intubation, which could be potentially harmful for the patient.

The introduction of S100B sampling in the ER has introduced a way to potentially screen which patients are in need of a CT scan. A meta-analysis on mild TBI and S100B, including 2,466 patients from 12 articles, states that the pooled negative predictive value (NPV) was more than 99% (CI 98%–100%) with a sensitivity of 97% in S100B detecting CT-visual brain pathology, using 0.10 μg/l as the cutoff [193]. This sensitivity and NPV make S100B superior to D-dimer, for example, in detecting pulmonary embolism and deep vein thrombosis (NPV: 92%) [198] and troponin-T (NPV: 96%) [60] in detecting myocardial infarction. The authors, however, do stress the importance of acquiring the sample early, within 6 h, after a mild TBI (due to the short half-life) and not using it in patients with extracranial injuries, since these patients will present S100B from extracranial sources and would thus lower the specificity for intracranial lesions [193].

This meta-analysis formed the foundation for the new Scandinavian CT guidelines for mild and moderate TBI from 2013 [192]. These guidelines today assist physicians and personnel in the ERs in several Scandinavian hospitals to better determine which mild TBI patients are in need of a head CT scan by acquiring a serum sample of S100B. If the concentration is less than 0.10 μg/l within 6 h after trauma and the patient is suffering from a mild TBI without extracranial trauma and other risk factors, not performing a head CT scan could be considered [192]. In theory, it is estimated that this could reduce the number of unnecessary CT investigations by about a third in the emergency departments with more effective resource allocation.

A risk that must be avoided and has been seen locally is to use S100B indiscriminately as a general screening tool to identify those in need of a CT, not considering the clinical indication and possible extra-cranial trauma-related peaks, as this may increase the number of CTs done instead of decreasing it. One should bear in mind though that not all cerebral pathology is visible on CT scans [93]; some lesions appear only on magnetic resonance imaging (MRI) or the more accurate diffuse tensor imaging (DTI). Authors are suggesting that some of these “false-positive” S100B levels might indicate lesions only seen on MRI [72], and as more sensitive radiological techniques become available, perhaps the specificity for S100B to detect intracranial lesions will increase. Some of the CT scans in studies included in the meta-analysis that formed the basis of the Scandinavian guidelines [193] could also have been performed too early (within 90 min) and thus may have missed the natural progression of cerebral contusions [199]. An inherent risk we have noted when using S100B to identify patients with mild TBI requiring a CT scan in the ER is if instead it is used as triage prior to clinical evaluation. This will recruit an unintended cohort of patients often with trauma-related S100B levels, increasing the risk of false-positive results and the risk of unnecessary CT scans. As mentioned, another factor that may cause “falsely” increased S100B levels is darker skin [15], something that may need to be further evaluated and possibly incorporated into current guidelines.

Theoretically and shortly after injury, the sensitivity of identifying a brain injury requiring a CT scan using S100B with a relatively low cutoff (i.e., 0.1 μg/l) is high, as a substantial amount of S100B will be released from the brain, as well as from potential extracranial sources [182]; however, this specific cutoff also results in a low specificity of intracranial injury if sampled too early. As time progresses, the short serum half-life of S100B will lead to a high wash-out of S100B, especially if there is no ongoing intracranial release or progressing injury.

A recent retrospective external validation of the new Scandinavian CT guidelines from 2013 was published last year [194], where the authors noticed that in a cohort of 662 patients, the guidelines had a sensitivity of 97% and a specificity of 34%, and this resulted in a CT reduction of 32% if applied [194]. One patient had an S100B level <0.1 μg/l and a small cortical contusion, but this patient presented with a mild TBI and perhaps would not have had a CT performed if other guidelines had been followed. Furthermore, the patient fully recovered shortly after injury. Another recent report from Calcagnile and co-workers revealed that the implementation of S100B to screen for intracranial lesions led to a cost reduction of 39€ per patient in their department, with theoretical savings of up to 70€ per patient if implemented correctly according to the guidelines [27].

In summary, we conclude that, given the above caveats concerning specificity, sensitivity and usage, the current suggested cutoff levels appear to provide a reasonable and clinically useful tool to detect CT visible intracranial pathology in mild TBI patients. Given the introduction of the Scandinavian guidelines, the effects of this transition will however need to be monitored for safety.

## Potentially missed lesions in mild TBI because of the 2013 Scandinavian guidelines

Recently, a new meta-analysis was published including n = 3,893 patients [63], suggesting higher cutoff levels (0.16–0.20 μg/l) to improve the specificity (to 50.69%) to detect CT-visible intracranial pathology. However, as we approach 6 h after trauma, the lower cutoff of 0.1 μg/l is probably better as it appears to assure an acceptable level to detect injury. By choosing a higher cutoff, we increase the risk of missing epidural hematomas, for example, which often present with only marginally elevated S100B levels [189]. While Undén and co-workers did not find any patient with EDH to have an S100B lower than 0.14 μg/l [189], others have detected S100B levels well below 0.1 μg/l in these patients [5, 202]. However, in these studies the Scandinavian guidelines were not implemented, and the exact time after trauma was unknown, thus confounding interpretation of these results. As the cerebral parenchyma is only marginally affected in the early stages of a progressing EDH, lower brain biomarker levels are expected. Presumably, some of the early S100B contribution in these cases could come from associated cranial fractures [131].

That even 0.1 μg/l of S100B might miss patients with positive CT findings was highlighted in the external validations of the Scandinavian CT guidelines [194]; however, these guidelines appear to have a similar capability to discriminate those in need of CT scans as other CT guidelines for mild TBI used worldwide [171].

In summary, the S100B cutoff to detect intracranial pathology in mild TBI should not be used in isolation, but as an integrated part of the assessment including clinical evaluation in order not to miss potentially harmful lesions, in concordance with the Scandinavian guidelines.

## S100B to assess sports-related brain injuries

Another field where mild, and often repetitive, TBI is common is in contact sports. Here, the unreported incidence may be higher than 600 per 100,000 people per year compared to the 200 per 100,000 reported incidence [29]. While a minimal number of these accidents results in verifiable cerebral lesions that need extensive medical attention, several athletes suffer from post-concussion-like symptoms (including chronic traumatic encephalopathy, CTE [126]) affecting somatic, affective and cognitive capabilities, sometimes persistent, preventing return to work and other daily activities [29].

Several studies have highlighted the role of S100B as a marker of brain injury severity in this group that was recently reviewed [129, 161], where S100B levels, among other things, were correlated to the ability to return to work [174]. Again, it should also be noted that S100B is increased in athletes without any obvious head injury, such as swimmers and marathon runners [161], which could be due to extracranial release, representing a confounder especially in mild head injuries where a limited amount of brain parenchyma might be affected.

## Clinical use of S100B in children

Perhaps the area that may best benefit from a surrogate marker to rule out unnecessary CT scanning is the pediatric population, as radiation emanating from CTs has been shown to be particularly harmful in this group [24, 59]. In fact, for a 1-year-old child, studies estimate the risk of developing a lethal malignancy from a single CT of the brain to be as high as 1:1500 compared with 1:5000 for a 10-year-old child [24]. Additionally, S100B is neurotrophic, and levels in serum are generally increased in the pediatric population with a growing CNS, thus presenting great variation of normal reference levels up to 15 years of age [8, 21, 30]. While higher cutoffs have been suggested for 0–24 month olds (0.35–0.23 μg/l) [22], the Scandinavian Neurotrauma Committee recently found it presently not possible to implement S100B using a similar cutoff approach as used in adults to detect patients eligible for CT scanning in children [9]. More knowledge of age-dependent normal values need to be evaluated before S100B can be used as a safe guide for decision making concerning CT scanning in children. Moreover, S100B is easy to obtain via both capillary and urinary samples, both representing ways to detect the protein [8, 18]. Not having to use venipuncture could be preferable for children. However, the utility of urinary S100B levels warrants further research and evaluation.

In more severe pediatric TBI cases, temporal profiles of subsequent samples of S100B in serum, referred to as a trajectory analysis, with increasing serum levels have a higher risk of unfavorable outcome compared to patients with subsequent serum concentrations in steady decline [17]. Thus, S100B may represent a similar surrogate marker of injury for outcome prediction and monitoring to detect secondary injuries as in the adult population. Other areas where S100B might provide important information about cerebral injuries are in the assessment of preterm newborns [163] and following hypoxic-ischemic injuries [53], recently reviewed respectively.

In summary, while S100B could benefit the pediatric population in the assessment of brain injury, more research is necessary before possible guideline implementation.

## S100B and outcome prediction in moderate-to-severe TBI in adults

Several reviews have analyzed how S100B may be utilized in moderate and severe TBI patients [16, 49, 66, 86, 92, 96, 130, 147, 162, 176, 186].

As severely brain injured patients enter the emergency department and the intensive care unit, they are often sedated and/or intubated making neurological assessments problematic. While CT examinations aid in the diagnosis and surgical planning, it is often difficult to fully predict the outcome of these patients [114]. Thus, a marker of brain tissue fate that may guide initial treatment and resources for patients in most urgent need in this critical phase is highly sought.

One of the latest reviews of S100B from 2013 includes a meta-analysis of 39 studies, including a total of 1,862 patients [104]. The authors found that serum levels between 2.16 μg/l and 14.0 μg/l predicted an unfavorable outcome, defined as a Glasgow Outcome Score (GOS) of 1 (death), 2 (vegetative state) or 3 (severe disability), and similar serum levels in six of the included studies were associated with mortality [104]. This wide concentration range is a huge problem for standardization and accurate cutoffs for outcome prediction. One of the major reasons for this, which is also discussed by the authors of the review, is the difference in sampling time in the studies. The optimal time for collecting S100B to predict outcome after trauma in moderate-to-severe TBI has been a matter of intense discussions. In several studies, the initial sample of serum S100B after trauma is considered the most important for outcome prediction [77, 136, 147, 152]. Later time frames have also been suggested to have clinical significance, ranging from within 6 h [201], 6 to 12 h [147], within 12 h [112], after 12 h [145], 24 h [50], within 42 to 79 h [67], within 48 h [134, 197], 72 h [113] and finally up to >84 h after trauma [134]. Thus, the serum concentration of S100B itself is of limited use to predict outcome if the time that has lapsed since the trauma is unknown.

**Fig. 1:**
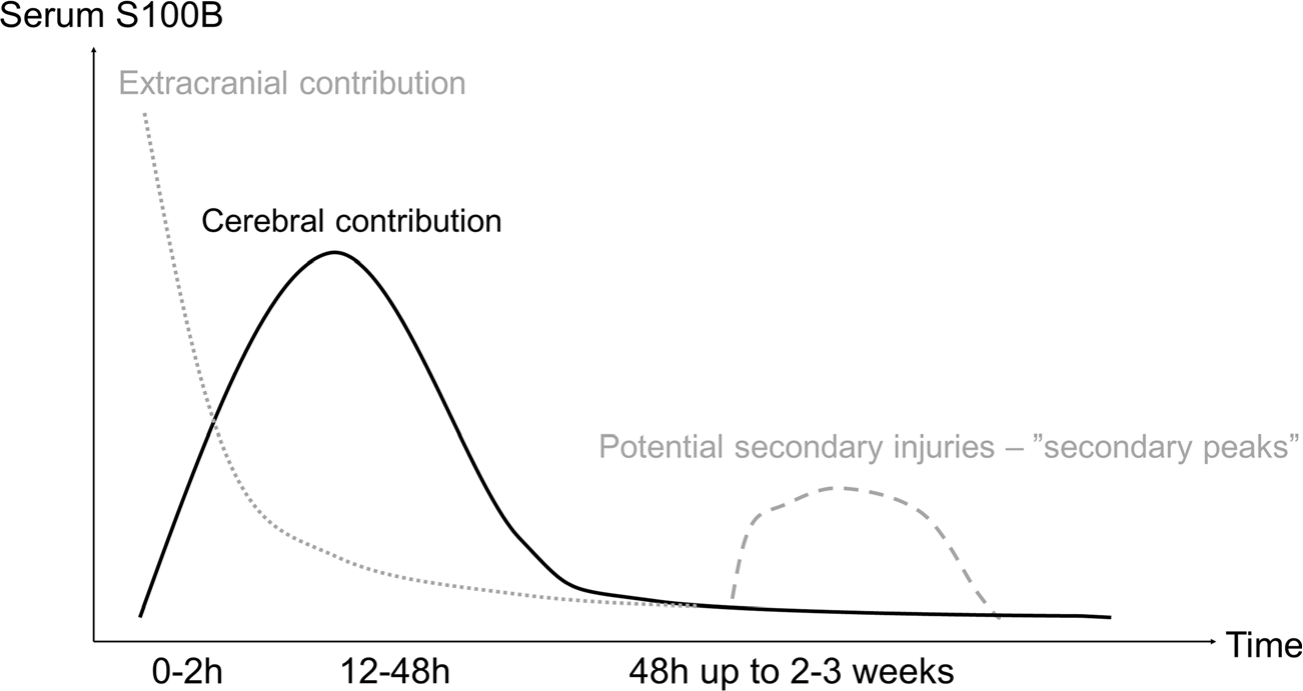
Schematic overview of S100B release to serum. Schematic overview of the S100B release following severe TBI. Initially, there will be a great release of S100B from extracranial tissue to the serum (*dotted, gray line*), which will have a rapid wash-out the first hours after injury. While the cerebral release is more prolonged and shaped as a gamma function, as suggested by Ercole et al. 2016 (black line), it will initially be “masked” by these extracranial contributions. Our *black line* illustrates an “uneventful” release of S100B in a patient suffering from severe TBI; however, patients may suffer from subsequent injuries resulting in “secondary peaks” of S100B (*dashed, gray line*)

Our group has shown, in a cohort of 265 NICU TBI patients, that the initial serum sample of S100B at admission has lower predicted accuracy toward outcome compared to later samples acquired approximately 24 h after admission [180]. The predictive accuracy of S100B is in fact 2–3× higher than age, pupil responsiveness or GCS at admission separately, all being known independent outcome predictors of injury suggested by the IMPACT (International Mission for Prognosis and Analysis of Clinical Trials in TBI) study group [114]. In a more recent study from our group, including the largest single-center patient cohort to date (n = 417 NICU TBI patients), the best outcome prediction for S100B occurred at about 30 h after reported trauma, with pseudo-explained variance of about 25% [179]. This latter predictive value is in congruence with our findings of a fitted kinetic model designed by our group, where the peak was found to be at 27 h [42]. In Fig. 1, we present a schematic overview of how we interpret the temporal release patterns of S100B and sources involved. The reason for the weak initial correlation between early S100B and outcome appears to be the result of the extracranial contribution of S100B “masking” the intracranial release. At 30 h, patients with a long-term GOS 1 (death) had a median S100B of 1 μg/l, GOS 3 (severe disability) 0.5 μg/l, GOS 4 (moderate disability) 0.3 μg/l and GOS 5 (good recovery) 0.25 μg/l, while it was not possible to detect specific serum levels significant for different GOS scores at admission [179].

The capabilities of S100B to predict outcome have led some authors to conclude that S100B could be used as a marker of brain death following injury [41, 164]. However, we believe that there are several reasons why this is potentially dangerous and should be avoided. First, due to its low specificity of brain injury, other reasons for S100B increases should always be explored. We have for instance seen extreme increases of S100B in serum [135] in a patient with mild TBI and malignant melanoma. Second, the S100B levels suggested by authors promoting S100B as a marker for brain death diagnostics (0.372 μg/l the first 24 h) are levels in patients that we repeatedly have seen return to a favorable outcome 12 months after injury [180]. Third, we hypothesize that S100B needs perfusion through the affected tissue for it to be released to the blood and that this process will be non-existent in brain dead patients, making S100B levels unreliable. Our clinical experience, having followed S100B in close to 3,000 NICU patients (both TBI and subarachnoid hemorrhage patients), is that levels prior to verified brain death are highly variable. Undén and co-workers measured S100B during this condition and noticed that without adequate circulation (CPP, cerebral perfusion pressure) to the brain, because of a high ICP, considerably lower amounts of S100B will be released [191]. Hence, this is also probably why S100B is a poor biomarker in the early stages of thrombo-embolic stroke [45]. Our clinical experience also leads us to suspect that sharp S100B peaks after stroke may represent a reperfusion washout; however, this needs to be confirmed. Thus, S100B should not be considered as a tool to identify brain death or support decision-making concerning withholding of treatments in severe TBI cases.

In summary, after accounting for its limitations concerning the low specificity of early samples, S100B appears to be an important and useful predictor of functional outcome in moderate-to-severe TBI, stressing however that no compound prediction models in TBI to date are accurate enough to support end of life decisions per se. Instead, S100B should help physicians prioritize resources to patients and may be part of future patient stratification strategies.

## S100B as a monitoring marker of ongoing injury in adults

Studies that subsequently sampled S100B during the hospital stay described scenarios where the levels of S100B continue to increase following TBI [85, 134], and these “climbers” have been shown to present an unfavorable outcome [177]. Moreover, patients without a steady decline of S100B in CSF and serum have been shown to present with worse outcome [51, 121, 149]. Serial S100B has even been suggested to outperform repeated radiological examinations to predict outcome [52]. Thus, there is a clinical interest in studying the kinetics of S100B following traumatic brain injury.

As the half-life of S100B in serum is short, a recent serum increase would theoretically correspond to a recent cerebral injury. We carefully mapped the kinetics of S100B in NICU TBI patients and noted that even small changes in time dramatically alter S100B levels and that the peak in the serum S100B concentration with seemingly unaffected wash-out is around 27 h after reported trauma [42]. Moreover, deviations from this normal curve as well as secondary peaks of S100B (Fig. 1) would presumably be a result of ongoing or new cerebral lesions.

**Fig. 2:**
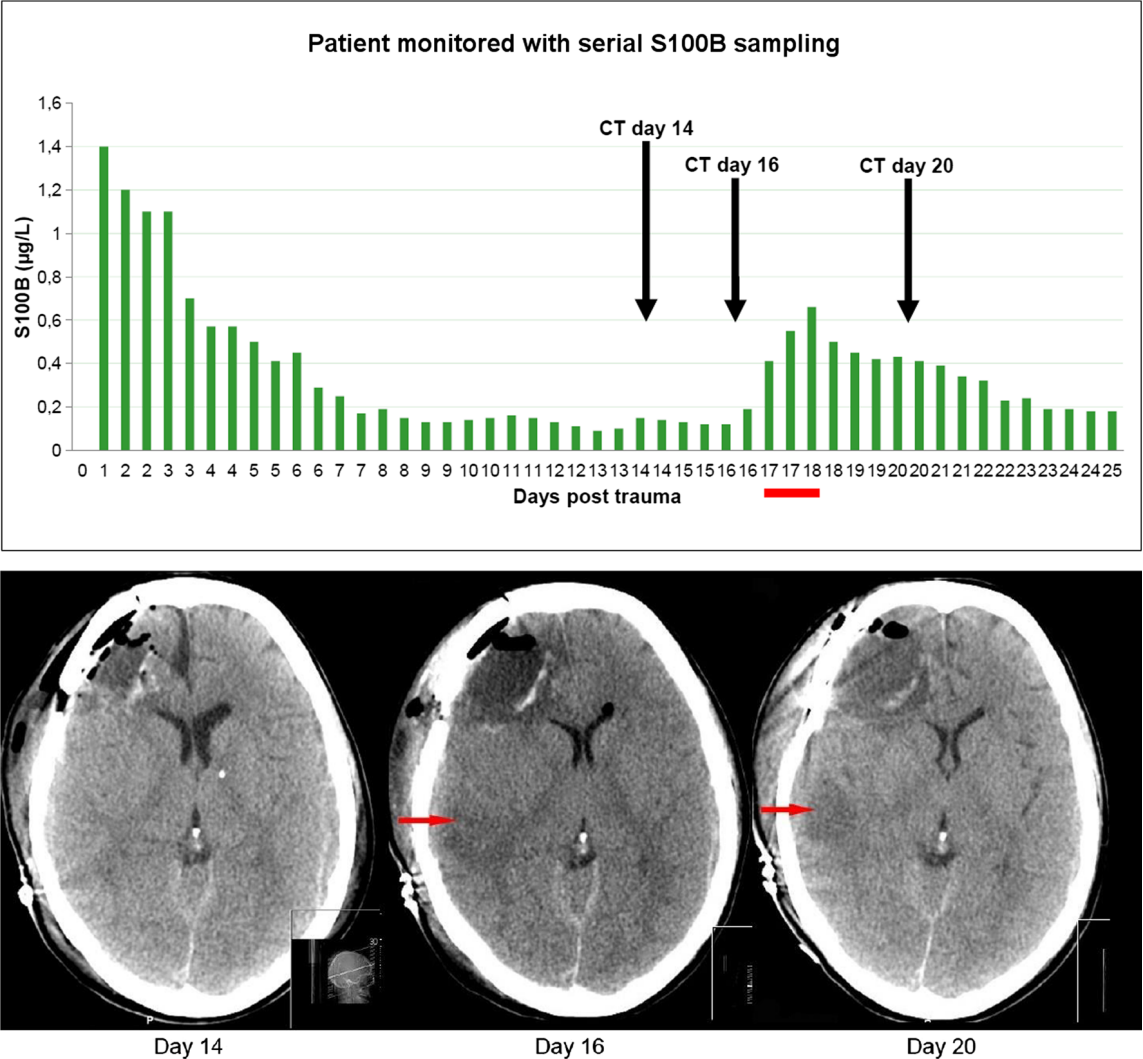
S100B monitoring of a TBI patient. The patient was monitored with subsequent sampling of S100B (twice per day, y-axis μg/l of S100B and x-axis days after trauma) illustrating an initial decline the first days following trauma approaching baseline levels. However, at day 16–18 (*bar*), there is a secondary peak of S100B that correlates to the development of a right temporal infarction as seen on computerized tomography (*arrows*)

Raabe and co-workers took daily S100B samples from their patients and found that a secondary increase of S100B >0.5 μg/l significantly correlated to the development of a severe secondary injury, such as a cerebral infarction or hematoma progression [148]. Moreover, they found that this secondary increase of S100B influenced treatment and diagnosis in 21% of the cases. An additional study by Undén et al. revealed that increased levels of S100B were correlated with secondary neurological complications [188], but the authors could not demonstrate a robus correlation between the secondary peaks and these complications, or that these complications were associated with long-term outcome. We noted in a cohort of 250 NICU TBI patients that a secondary increase as subtle as 0.05 μg/l in patients sampled twice daily had a sensitivity of 80% and a specificity of 89% to detect secondary radiological pathological findings [181]. Moreover, these secondary increases in S100B were significant predictors of unfavorable outcome. In Fig. 2, we illustrate how serial sampling of S100B detected a secondary peak of S100B, which correlated to the development of a right temporal infarction, a deterioration not detected using ICP monitoring. Our findings are similar to those of other TBI cohorts; several studies have shown a correlation between neurological deterioration and a secondary increase of S100B during the NICU stay, suggesting it to be a useful therapeutic tool closely related to pathophysiological mechanisms in TBI [35, 65, 90].

We have shown that serum S100B has definable kinetic properties following TBI [42]. We are of the conviction that kinetic properties are a key strength of S100B as compared to certain other markers, such as GFAP, while GFAP, presumably because of its higher brain specificity, may be advantageous compared to S100B in screening mild TBI patients who require a CT in the ER [131]; the half-life of GFAP in serum is significantly longer than that of S100B (25 min vs. <48 h) [34, 79]. Thus, S100B should theoretically wash out quickly from the system if no S100B is contributed from an ongoing or evolving cerebral lesion, as it contains so much more S100B compared to other tissues. This is the primary reasons why it is difficult to establish outcome thresholds for S100B levels, as they will change rapidly in the serum during the first days following TBI [179], but at the same time it is this dynamic information content that strengthens their potential role as a monitoring marker and supports serial sampling [148, 181]. Therefore, using kinetic modeling, we defined the gamma-variate curve that S100B is expected to follow [42]. This allows for reliably estimating the projected peak from any time point given that the time of trauma is known and provided that no secondary insults initiate a new release sequence.

In summary, these findings strengthen the role of S100B as a laboratory test for monitoring patients admitted to the NICU with frequent sampling preferable to detect potential injury progression that may prompt diagnostic and therapeutic actions.

## S100B as a surrogate marker for treatment efficacy

Several groups, including ours, have found a correlation between ICP and serum S100B levels [14, 62, 124, 134, 181]. Since ICP is currently the most widely used surrogate marker to assess intracranial conditions, it supports the theory of S100B as a marker to guide treatment in the NICU.

Preclinically, S100B has been shown to be useful in determining the success of ischemic stroke treatment in rabbits [203]. Clinically, S100B has been used as a marker to validate the treatment effect in TBI patients between hypertonic saline-dextran (HSD) and normal saline, where the HSD group presented lower levels, presumably indicating a less affected brain parenchyma [11]. During procedures such as hypothermia treatment following cardiac arrest [110], carotid endarterectomy [3] and cardiac surgery [187] and hyperbaric oxygen therapy following TBI [178], S100B levels in serum may be used to detect and treat any ongoing cerebral injury. Moreover, in clinical depression, S100B has been shown to be able to predict treatment response [4] and extra-cranially, as mentioned, to follow the treatment of malignant melanoma [40].

Currently, there are no pharmaceutical treatments to date that specifically target the underlying pathophysiology in TBI as all large placebo controlled randomized control trials have failed to show significant treatment efficacy [61], including, e.g., methyl-prednisolone [150], progesterone [205] and erythropoietin [119]. One of the reasons these trials have failed is believed to be a lack of methods to validate the therapeutic effect and instead focus predominantly on late outcomes, which may be influenced by a multitude of factors, thus introducing noise and possibly drowning possible treatment effects. In a recent, smaller study of erythropoietin in human TBI, S100B was used as a proxy of treatment effect with decreasing levels seen in patients treated with the drug vs. placebo, thus suggesting a lesser extent of tissue injury [94].

In summary, by introducing serum biomarkers such as S100B in future pharmaceutical trials, as well as better radiological markers [28], it may be possible to better monitor surrogate markers of effect in relation to specific pharmacological treatments.

## Other protein biomarkers of brain tissue fate

There are several other theoretically brain-specific proteins that are used as biomarkers to assess brain injury [206]. Here, we focus briefly on some of the most studied in the context of S100B.

## Neuron-specific enolase (NSE)

The second most published biomarker of brain injury is NSE. NSE is an iso-enzyme of enolase located primarily in the cytoplasm of neurons, involved in glycolysis by transforming 2-phosphoglycerate into phosphoenolpyruvate. When released in blood, it has a half-life of approximately 24 h in patients not affected by brain injury, to 48 h in patients suffering from brain injury [73, 78, 160]. Recent reviews and meta-analyses highlight its properties as an independent marker of functional outcome and mortality [31], albeit it being inferior to S100B [105]. The main limitation of NSE is its presence in red blood cells, causing problems in serum analyses in the case of hemolysis [46]. While modern techniques may try to adjust for this [185], hemolysis will often cause “false increases” of NSE making it difficult to assess in the emergency and NICU setting. In our group, we recently compared S100B and NSE and found that S100B was a better outcome predictor overall, with NSE only predicting mortality [179]. Importantly, despite NSE originating from neurons as opposed to astrocytes, a high covariance between S100B and NSE was seen. This also explains why it did not provide any additional information over S100B toward outcome prediction. Moreover, while an extracranial contribution from multitrauma was evident for S100B, this normalized within 12 h. However, the correlation of NSE levels to multitrauma extends past 72 h. This could theoretically be due to its longer half-life, but also a contribution of NSE from trauma-related hematomas is possible [179].

## Glial fibrillary acidic protein (GFAP)

GFAP is an intermediate filament cytoskeleton protein primarily found in astrocytes; thus, it shares an origin with S100B. However, compared to S100B, GFAP is more brain specific [133, 166], which is probably why one study found a greater specificity in detecting CT-verifiable lesions in mild TBI patients compared to S100B in the presence of extracranial injury (5% vs. 55%) [131]. While kinetic studies have revealed a prolonged release and higher levels in mild TBI patients with CT lesions [127], no definitive results exist on the in vivo half-life. In vitro studies put the half-life of GFAP somewhere between 16–144 h for different subunits [32, 151, 169], while one clinical study suggested it is <48 h in serum [34]. Although GFAP has been shown to be elevated in patients needing neurosurgical intervention [127], it has not been as extensively studied as a monitoring marker as S100B. For outcome prediction in moderate-to-severe TBI, GFAP has been shown to exhibit similar outcome predictive capabilities as S100B, and a combination of the two markers may improve outcome prediction models [132, 204]. In summary, while GFAP is a promising marker of brain injury in both mild and moderate-to-severe TBI, its kinetic profile and outcome predictive effect are less studied compared to S100B’s.

## Ubiquitin C-terminal hydrolase L1 (UCH-L1)

One of the newer serum brain biomarker candidates is UCH-L1. UCH-L1 is a deubiquitinating enzyme modifying the c-terminal adduct of the ubiquitin monomer present primarily in neurons. Pre-clinical studies have been very promising with increasing levels early after both TBI and stroke [95]. The half-life in serum has been shown to be 11 h in TBI patients [26]. In clinical studies, results show that it may be used to screen for CT-verifiable lesions in mild TBI, but presents less specificity than many studies using S100B [128, 143] and does not provide the same accuracy over time to assess brain injury as GFAP [127]. Moreover, in moderate-to-severe TBI, while UCH-L1 levels have been shown to predict outcome, they do not add significant independent information to more extensive outcome models [142].

## Neurofilament light (NF-L)

Neurofilaments, the main components of the axonal cytoskeleton, consist of three chains, where the protein NF-L constitutes the smallest (68 kDa). CSF levels of NF-L are used as a biomarker in multiple sclerosis to monitor effect of treatment [55]. There are fewer studies on TBI, but it shows promising results as a potential marker of injury severity as CSF samples have been correlated to the extent of injury sustained during boxing [118]. The serum half-life of NF-L is presumably long, suggested to range up to 3 weeks [12]. In our group, we have shown that the serum level of NF-L is a predictor of outcome in moderate-to-severe TBI and adds independent information significantly different from S100B [2]. This is perhaps not surprising as it represents a very different tree of TBI pathophysiology than S100B. However, this biomarker is not particularly dynamic in the early TBI stages and may not be helpful for early prediction and monitoring purposes [2].

## Conclusions

S100B levels in serum are a useful marker of brain tissue fate in TBI. Several difficult clinical situations, such as determining the need for CT scanning in mild TBI, monitoring unconscious TBI patients, predicting outcome and validating treatment effect, may be facilitated by the use of S100B. The emerging understanding of the kinetics of S100B may help avoid pitfalls and increase its utility. Despite its shortcomings, and if appropriately used, S100B is a valuable and yet underused tool for clinicians managing TBI patients. Future research should focus on understanding and discriminating the origin of ultra-early and later levels of S100B and on defining age-related reference values for the pediatric population. Furthermore, given the strong predictive value for outcome in more severely injured TBI patients, we suggest that future novel biomarker studies should be compared to S100B in multivariate analyses including other known predictors, such as the IMPACT variables.
